# Antiulcer Activity of Indigenous Plant *Operculina turpethum* Linn.

**DOI:** 10.1155/2013/272134

**Published:** 2013-02-11

**Authors:** Vidya Ignatius, Madhusudhanan Narayanan, Venkataraman Subramanian, Balasubramanian Maruthaiveeran Periyasamy

**Affiliations:** ^1^Department of Pharmacology, Sathyabama University Dental College and Hospital, Sathyabama University, Jeppiaar Nagar, Rajiv Gandhi Road, Chennai, Tamilnadu 600 119, India; ^2^Department of Pharmacology and Environmental Toxicology, Dr. ALM Post Graduate Institute of Basic Medical Science, University of Madras, Taramani Campus, Chennai, Tamilnadu 600 113, India

## Abstract

In the Indian traditional system of medicine *Operculina turpethum* is commonly used to treat various ailments including peptic ulcer, inflammation, and pain. Ulcer preventive and ulcer protective activities of HAOP and MOP stem bark extracts of *Operculina turpethum* (100 mg/kg, b.w., orally) were evaluated employing aspirin + pylorus ligation (APL) model in experimental rats. The results suggested that both extracts (HAOP and MOP) possess enhanced ulcer preventive and protective activities when compared with the standard drug ranitidine. HAOP showed more pronounced effect when compared to MOP. Further the result of the histopathological and biochemical studies also confirms potent ulcer preventive and protective nature of a extracts in a similar manner.

## 1. Introduction

Peptic ulcer is one of the common disorders of gastrointestinal tract, which occur due to an imbalance between the offensive (gastric acid secretion) and defensive (gastric mucosal integrity) factors [1]. Stress, smoking, nutritional deficiencies, and frequent intake of nonsteroidal-antiinflammatory drugs (NSAIDs) develop the peptic ulcer prevalence in the world [2]. It is a known fact that well-targeted therapeutic approaches are needed for the treatment of peptic ulcer disease.

A wide range of drug is currently available for treatment of gastric ulcer which includes proton pump inhibitors, H_2_-blockers, antiacids, and anticholinergics. The most common adverse effects of these drugs are hypergastrinemia, hypersensitivity, gynecomastia, impotence, arrhythmia, and blood dyscrasias such as thrombocytopenia and enteric infections (*Clostridium difficile*) [3]. These effects are the rationale for the development of new antiulcer drugs. For this reason, the hunt is still on to discover a natural medicine having antiulcerogenic properties.

A number of medicinal plants have been used for thousands of years in the traditional system of medicine (Ayurveda) to treat various diseases like peptic ulcer, cancer, diabetes, arthritis, hepatitis, acute and chronic inflammations, neurodegenerative diseases, and so forth [4].

*Operculina turpethum* belongs to the family Convolvuceacea [5, 6]. It is popularly known as “transparent wood rose” and widely used for the treatment of ulcers, tumors, neurological disorders, constipation, dysmenorrhea, and inflammation [7–9]. It is one of the components of Ayurvedic formulations such as *Trivrit choornam, Trivritadi choornam, *and *Avipattikar ghritam*.

It is a perennial climber and it exudates a milky juice. Stem is very long, twining and much twisted, angled, and winged. The roots are long, slender, fleshy, and much branched. The black variety of root is powerful drastic, bitter with a sharp sweet taste while white variety is moderately cathartic and red variety is sweetish acrid [5, 6].

The plant root contains a glycoside resin, which is mainly concentrated in the root bark. It contains glycosides (Scopoletin, turpethinic acid A, B, C, D, and E). Stem of the plant contains triterpenes (betulinic acid, betulin, and lupeol) and sitosterol [5, 10, 11]. There are a few reports on the cooccurrence of both calystegines and swainsonine, a polyhydroxylated indolizidine alkaloid well-known from certain convolvulaceous species [12]. The plant possesses anticancer, antiproliferative [13], antimicrobial [14], antidiabetic [15], anti-inflammatory [16], and antihepatotoxic [17, 18] activities.

Though the Plant has been extensively for various diseases including treatment of ulcers. Hence, an attempt has been made to screen the ulcer preventive and protective activity of the extracts of the stem bark of *Operculina turpethum* in animal model.

## 2. Materials and Methods

### 2.1. Plant Material

The dry stem of *Operculina turpethum* was purchased from the Indian Medical Practitioners Co-operative Society (IMCOPS) Chennai, Tamil Nadu, India which was authenticated by Dr. S. Jayaraman, Director at the Plant Anatomy Research Center, Chennai, Tamil Nadu, and a voucher specimen has been deposited at the herbarium unit of the Department of Pharmacology and Environmental Toxicology, University of Madras, Taramani, Chennai.

### 2.2. Preparation of Extracts (Methanolic (MOP) and Hydroalcoholic (HAOP))

*Operculina turpethum *stem bark was scrapped, chopped, shade dried and coarsely powdered. One portion of the powder was then extracted with methanol using Soxhlet extractor and the other portion was extracted with ethanol-water in the ratio of 1 : 1, being stirred and macerated at room temperature for 10 days. The extracts were dried under reduced pressure using rotary flash evaporator and stored at −20°C. The percentage yields of hydroalcoholic (HAOP) and methanolic extracts are 9% w/w and 6% w/w, respectively.

### 2.3. Animals

Forty-two male Wistar Albino rats (150 ± 20 g) procured from the Tamil Nadu University of Veterinary and Animal Science were used for the study. The animals kept in polypropylene cages were acclimated to laboratory conditions for one week before starting the experiment (12 h dark : 12 h light cycles; 22 ± 2°C) and provided with standardized pellet feed and water *ad libitum*. Animals that were submitted to oral administration of extracts and standard drugs were fasted for 18 h before the experiments. The experimental protocol was approved by the Institutional Animal Ethical Committee (IAEC NO: 07/042/05) and the study was conducted in accordance with CPCSEA guidelines.

### 2.4. Aspirin + Pyloric Ligation Induced Ulcer Model

#### 2.4.1. Experimental Setup

The animals were divided into seven groups of six animals each.

Group I: PL control animals
Group II: aspirin + pylorus ligated control animals
Group III: ranitidine (50 mg/kg/p.o) + pylorus ligated animals
Group IV: HAOP alone (100 mg/kg/p.o) treated animals
Group V: HAOP (100 mg/kg/p.o) + aspirin + pylorus ligated animals
Group VI: MOP alone (100 mg/kg/p.o) treated animals
Group VII: MOP (100 mg/kg/p.o) + aspirin + pylorus ligated animals.

The methods of the Goel et al. [19] and Shay et al. [20] were followed for the evaluation of preventive and ulcer-protective activities.

The groups III–VII received the assigned drug treatment for 10 days daily. From day 8–10, groups II, III, V, and VII animals received aspirin orally as an aqueous suspension (200 mg/kg) 2 h after administration of the drugs [19]. Animals in all the groups were fasted for 18 h and pylorus ligation was carried out on the 11th day.

Pylorus ligation was carried using light ether anesthesia; the abdomen was opened through a midline incision. Pylorus was exposed and ligated using fine thread around it without disturbing the stomach. Then animals were sutured and returned to their cages, respectively. Four hours after pylorus ligation, the animals were sacrificed by cervical decapitation.

The stomachs were removed and opened along the greater curvature and washed; gastric lesions were observed. The gastric juice collected by a modification of the pylorus ligation technique described by Shay and his colleagues [20, 21] is a valid approach for collection of gastric juice in the rat models. Then the samples were subjected to biochemical analysis; the volume of the supernatant was expressed as mL/100 g body weight [22]. Ulcers were scored and the ulcer index was determined [23]. Total acidity, free acidity, and various biochemical investigations were also carried out, respectively.

### 2.5. Statistical Analysis

Data are presented as the mean ± standard error (SEM). One-way ANOVA followed by Tukey's multiple comparison test was used to compare the mean of different groups by using SPSS 7.5 Student version.

## 3. Results

### 3.1. Biochemical Findings

#### 3.1.1. Ulcer Index and Percent of Protection

The ulcer index and percent of protection against ulcers in the pylorus ligated model are shown in Tables 1(a) and 1(b). Treatment with MOP and HAOP showed significant protection against ulcers in pretreatment (76.53% and 81.32%) and posttreatment (54.21% and 60.13%) at a dose of 100 mg/kg (*P* < 0.001) when compared with the control animals, respectively. The standard drug, ranitidine, showed significant protective effects against ulcers (65.14%) at a dose of 50 mg/kg when compared with the control groups (*P* < 0.001) in both treatments.

#### 3.1.2. Free Acidity, Total Acidity, Total Acid Output and Gastric Volume

**Figure 1:**
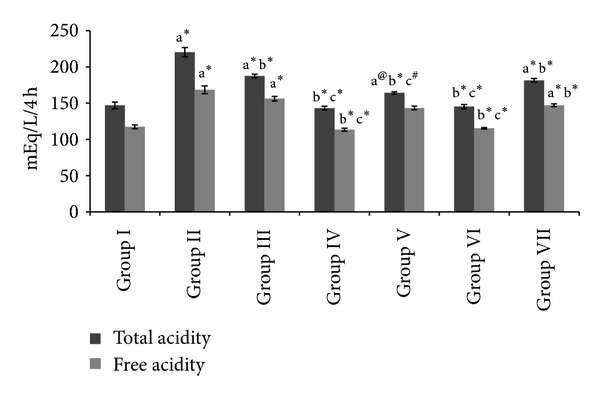
Effect of HAOP and MOP extracts of *Operculina turpethum* on total acidity and free acidity in aspirin + PL induced ulcers. Values are expressed as mean ± SEM of 6 animals. Comparisons were made between (a) Group I versus II, III, IV, V, VI and VII, (b) Group II versus III, IV, V, VI, and VII, and (c) Group III versus V and VII. Symbols represent statistical significance: *P < 0.001, ^#^ *P* < 0.01, and ^@^ *P* < 0.05.

**Figure 2:**
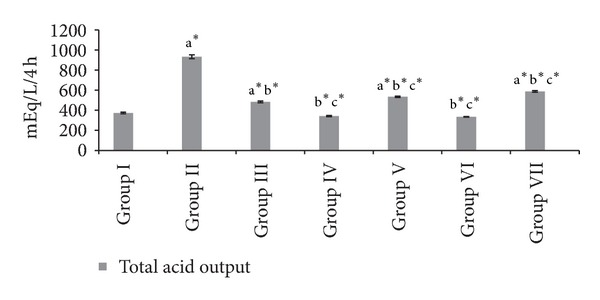
Effect of HAOP and MOP extracts of *Operculina turpethum* on total acid output in aspirin + PL induced ulcers. Values are expressed as mean ± SEM of 6 animals. Comparisons were made between (a) Group I versus II, III, IV, V, VI, and VII, (b) Group II versus III, IV, V, VI, and VII, and (c) Group III versus V and VII. Symbols represent statistical significance: *P < 0.001, ^#^ *P* < 0.01, and ^@^ *P* < 0.05.

**Figure 3:**
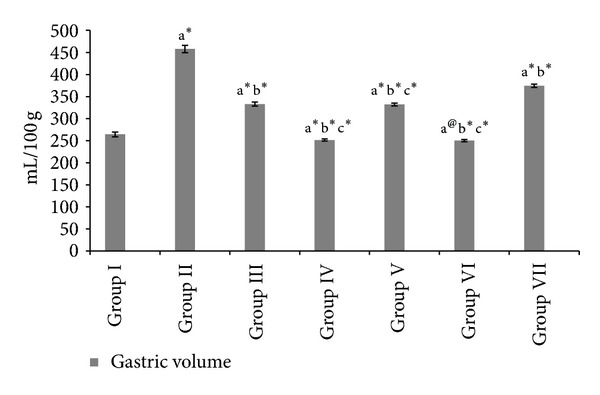
Effect of HAOP and MOP extracts of *Operculina turpethum* on gastric volume in aspirin + PL induced ulcers. Values are expressed as mean ± SEM of 6 animals. Comparisons were made between. (a) Group I versus II, III, IV, V, VI, and VII, (b) Group II versus III, IV, V, VI, and VII, and (c) Group III versus V and VII. Symbols represent statistical significance: *P < 0.001, ^#^ *P* < 0.01, and ^@^ *P* < 0.05.

Figures 1, 2, and 3 shows the free acidity, total acidity, total acid output, and gastric volume of the experimental groups. Administration of both the extracts and ranitidine showed a significant reduction (*P* < 0.001) in the total acidity, free acidity, total acid output, and gastric volume.

#### 3.1.3. Mucin Activity, Protein, and Total Carbohydrates Protein Ratio

**Figure 4:**
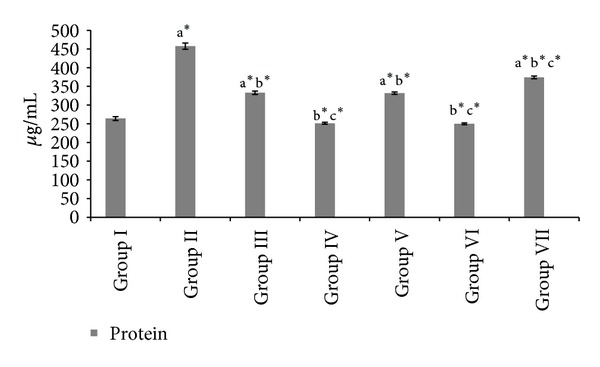
Effect of HAOP and MOP extracts of *Operculina turpethum* on protein in aspirin + PL induced ulcers. Values are expressed as mean ± SEM of 6 animals. Comparisons were made between (a) Group I versus II, III, IV, V, VI, and VII, (b) Group II versus III, IV, V, VI and VII, and (c) Group III versus V and VII. Symbols represent statistical significance: *P < 0.001, ^#^ *P* < 0.01, and ^@^ *P* < 0.05.

**Figure 5:**
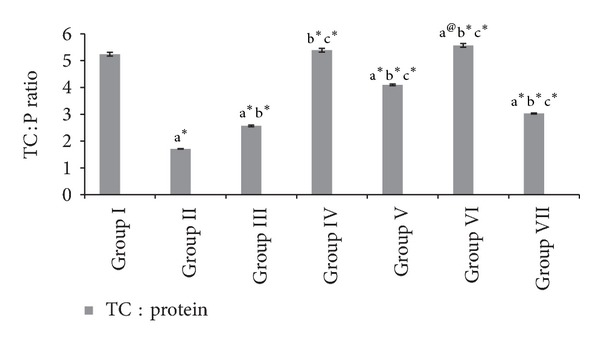
Effect of HAOP and MOP extracts of *Operculina turpethum* on TC : protein in aspirin + PL induced ulcers. Values are expressed as mean ± SEM of 6 animals. Comparisons were made between. (a) Group I versus II, III, IV, V, VI, and VII, (b) Group II versus III, IV, V, VI, and VII, and (c) Group III versus V and VII. Symbols represent statistical significance: *P < 0.001, ^#^ *P* < 0.01, and ^@^ *P* < 0.05.

Table 2 and Figures 4 and 5 shows the values of individual carbohydrates like hexose, hexosamine, sialic acid, fucose, total carbohydrates, protein, and total carbohydrates-protein ratio of the gastric juice in experimental groups. HAOP and MOP pre- and posttreatment showed a significant increase (*P* < 0.001) in the defensive mucin secretion of the gastric juice. Moreover protein concentration in gastric juice was significantly decreased with a significant increase in the total carbohydrate : protein ratio (TC : P) in the treated groups.

#### 3.1.4. Macroscopic Examination

**Figure 6:**
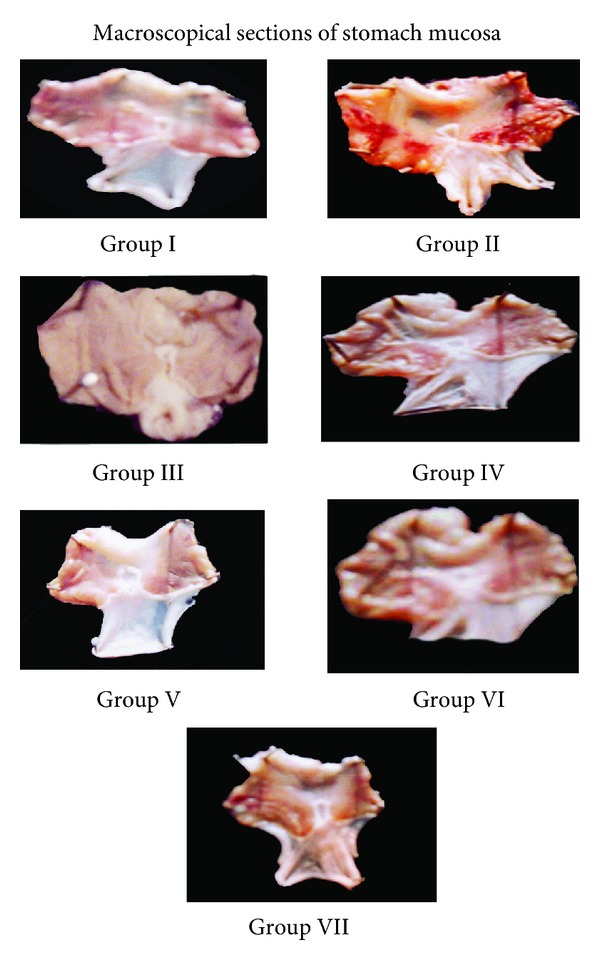


Macroscopic sections of stomach reveal the ulcer score and comparative effect of treatment with the extracts HAOP and MOP when compared with group II pylorus ligated animals visually (Figure 6).

### 3.2. Histopathological Changes

**Figure 7:**
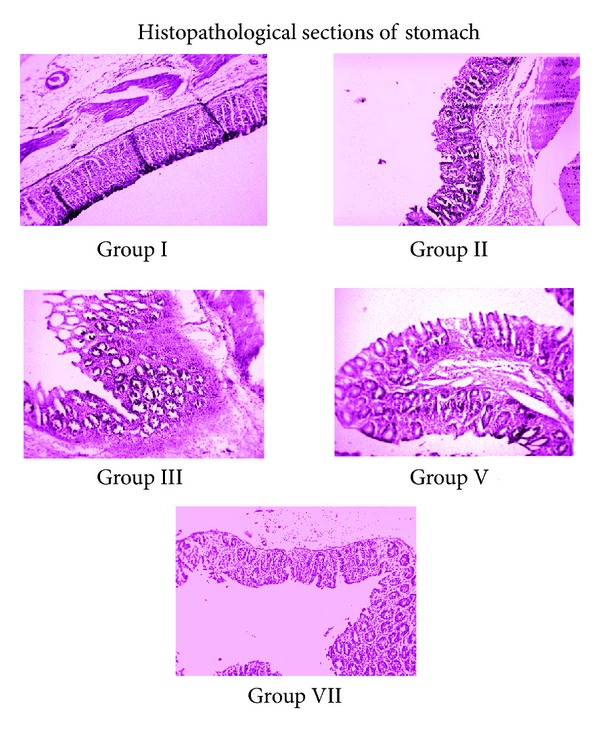


Histopathological examination of stomach mucosa shows that pretreatment with HAOP, MOP and Ranitidine protects the mucosal epithelium from the damage caused by aspirin. In HAOP and MOP treated groups the mucosa was found to be almost normal with mild edema on the lamina propria. Ranitidine treated section shows normal mucosa with no ulcer but with slight congestion (Figure 7 and Table 3).

## 4. Discussion

Gastric ulcers arise due to various factors [24]. Even though the etiology of gastric ulcers is still debated, it is accepted that ulcers are caused due to net imbalances in mucosal offensive and defensive factors [25].

Common NSAIDS like aspirin cause damage to the gastric mucosa by interfering with prostaglandin synthesis. Furthermore, it results in generation of free radicals, imbalance in gastric secretion, elevated pepsin, protein content, and back diffusion of H^+^ ions into the gastric mucosa leading to necrosis and ulceration [26, 27]. Wide ranges of remedial drugs including medicinal plants that are used in the treatment of peptic ulcer inhibit the gastric acid secretion and provoke the mucus secretion by reversing the consequence of aspirin. In the present study, pre- and posttreatment with HAOP and MOP extracts attenuated the gastric volume, free acidity, total acidity, total acid output, and ulcer index thus showing the antisecretory property. These results suggest the possible involvement of prostaglandins and/or mucus in the antiulcer activity of the extracts.

Gastric wall mucus, an obligatory component of which is hexosamine, is thought to play an important role as a defensive factor against gastrointestinal damage [28]. The determined gastric wall mucus was used as an indicator for gastric mucus secretion, while mucosal hexosamine content was used as an indicator for gastric wall mucus synthesis [29]. Aspirin + pylorus ligation decreased the concentrations of all the individual carbohydrates and also the carbohydrate-to-protein ratio. Previous findings of Vasudeva et al. [30] and Sanmugapriya and Venkataraman [31] reported a similar decrease in accordance with the present study.

Ulcer induced group II animals showed increase in protein content of the gastric juice indicating gastric mucosal damage due to leakage of plasma proteins [32–34]. Both extracts showed decrease in protein levels, which indicates strengthening of the gastric mucosa, thereby restricting the entrance of the plasma proteins into gastric juice. In addition a significant increase in the defensive mucin secretion was quantified in terms of hexose, hexosamine, sialic acid, fucose, and total carbohydrates : protein (TC : P) ratio of the gastric juice [35].

The increased mucus secretion in the extract treated groups may attribute to lessening of stomach wall friction during peristalsis and gastric contraction, improving the buffering of acid and by acting as an effective barrier to back diffusion of hydrogen ions [36]. Moreover, mucus is capable of acting as an antioxidant, and thus can reduce mucosal damage mediated by free radicals [37].

Further, histopathological examination of stomach mucosa shows that pre- and posttreatment with HAOP, MOP, and ranitidine protects the mucosal epithelium from the damage caused in the ulcer model. In HAOP, and MOP treated groups the mucosa was found to be almost normal with mild muscularis mucosa. Ranitidine treated section shows normal mucosa with no ulcer; edema in the submucosa establishes ulcer preventive and protective nature of the plant extracts.

The nonspecific gastroprotective findings of MOP and HAOP may be due to presence of phytoconstituents like triterpenoids (betulinic acid, epibetulinic acid, betulin, and lupeol), steroids (sitosterol), and glycosides (Scopoletin, turpethinic acid A, B, C, D, E) [5, 10, 11]. The mechanism of action of these phytochemicals may be attributed to causing the inhibition of gastric acid secretion or to boosting the mucosal defense mechanism by increasing mucus production, stabilizing the surface epithelial cells or interfering with the PGs synthesis.

Gastric acid secretion is influenced by histamine release, gastrin secretion, and acetylcholine release [38]. H_2_ blockers like cimetidine, ranitidine, and famotidine exhibit antihistaminic effect thus causing reduction in gastric acidity and secretory volume since they have a five-membered ring structure with a flexible side chain attached to polar uncharged group. Evidently, betulinic acid an active constituent in both the extracts having similar ring structure to H_2_ blockers may be a reason for the antihistaminic effect, which is responsible for gastroprotective nature [39, 40]. Moreover, betulinic acid, lupeol, betulin, and glycosides [31, 41–44] present in the extracts possess potent antioxidant properties by decreasing lipid peroxidation and increasing antioxidant levels.

On the other hand, the presence of flavonoids facilitates the increase in the mucosal prostaglandin levels and inhibition of histamine release thus exhibiting a protective effect of the extracts [45].

It is also reported that saponins is shown to have ulcer protective effect by the formation of protective mucus which shield the mucosa from acid damage by selectively inhibiting prostaglandins. Moreover, tannins precipitate the microproteins thereby forming an impervious protective pellicle over the ulcerated gastric lining resulting in inhibition of toxic substance adsorption and prevent from further proteolytic enzyme degradation [46].

In conclusion, the ulcer preventive and protective activity demonstrated in the present study provides a strong support for the traditional use of this plant in the treatment of gastric and intestinal ulcers.

## Figures and Tables

**Table:** (a)

Pretreatment	Dose (mg/kg)	Ulcer index	Protection (%)
Aspirin + pylorus ligated control	—	4.39 ± 0.04 a*	—
Ranitidine	50 mg/kg/p.o	1.53 ± 0.02 a*b*	65.14
MOP	100 mg/kg/p.o	1.03 ± 0.009 b*c*	76.53
HAOP	100 mg/kg/p.o	0.82 ± 0.007 a*b*c*	81.32

**Table:** (b)

Posttreatment	Dose (mg/kg)	Ulcer index	Protection (%)
Aspirin + pylorus ligated control	—	4.39 ± 0.04 a*	—
Ranitidine	50 mg/kg/p.o	1.53 ± 0.02 a*b*	65.14
MOP	100 mg/kg/p.o	2.01 ± 0.02 a*b*c*	54.21
HAOP	100 mg/kg/p.o	1.75 ± 0.02 a*b*c*	60.13

The following values are expressed as mean ± SEM of 6 animals.

Comparisons were made between.

a: Group I versus II, III, IV, V, VI, and VII.

b: Group II versus III, IV, V, VI, and VII.

c: Group III versus V and VII.

Symbols represent statistical significance: *P < 0.001, ^#^ *P* < 0.01, ^@^ *P* < 0.05.

**Table 2:** Effect of hydroalcoholic and methanolic extracts of *Operculina turpethum *on mucin activity of gastric juice in aspirin + PL induced ulcers.

Groups	Hexose (*μ*g/mL)	Hexosamine (*μ*g/mL)	Sialic acid (*μ*g/mL)	Fucose (*μ*g/mL)	Total carbohydrates (*μ*g/mL)
Group I	919.00 ± 18.68	243.04 ± 4.94	69.54 ± 0.67	97.77 ± 1.33	1266.06 ± 39.90
Group II	521.70 ± 9.52 a*	138.72 ± 2.53 a*	28.34 ± 0.27 a*	93.62 ± 1.70	723.00 ± 14.69 a*
Group III	719.04 ± 9.82 a*b*	166.50 ± 2.27 a*b*	37.70 ± 0.36 a*b^@^	99.70 ± 2.02	933.72 ± 18.98 a*b*
Group IV	860.64 ± 8.31 a^@ ^b*c*	252.07 ± 2.43 b*c*	68.87 ± 0.94 b*c*	100.50 ± 0.97 b^@^	1274.20 ± 17.40 b*c*
Group V	736.84 ± 7.11 a*b*	200.44 ± 1.93 a*b*c*	55.96 ± 0.76 a*b*c*	99.97 ± 0.96 b^@^	1109.38 ± 15.15 a*b*c*
Group VI	877.97 ± 11.99 b*c*	258.90 ± 2.50 a^@ ^b*c*	71.82 ± 3.64 b*c*	101.34 ± 1.38 b^@^	1285.27 ± 17.56 b*c*
Group VII	670.14 ± 6.47 a*b*c^@^	187.19 ± 1.80 a*b*c*	50.09 ± 0.67 a*b*c*	98.83 ± 0.95	1075.39 ± 10.38 a*b*c^#^

The following values are expressed as mean ± SEM of 6 animals.

Comparisons were made between.

a: Group I versus II, III, IV, V, VI, and VII.

b: Group II versus III, IV, V, VI, and VII.

c: Group III versus V and VII.

Symbols represent statistical significance: *P < 0.001, ^#^ *P* < 0.01, ^@^ *P* < 0.05.

**Table 3:** Histopathological sections of stomach mucosa.

Groups		Histological observations on stomach mucosa
Group I	PL control	Ulcerated mucosa showing discontinuity in the lining epithelium with edematous submucosa
Group II	aspirin + PL Control	Ulcerated mucosa showing hemorrhage and discontinuity in the lining epithelium with exudates in the lumen with submucosal edema
Group III	Ranitidine + aspirin + PL	Normal mucosa with no ulcer, edema in the submucosa
Group V	HAOP (100 mg/kg/p.o) + aspirin + PL	Normal mucosa with, small atrophic glands, no edema, mild muscularis mucosa
Group VII	MOP (100 mg/kg/p.o) + aspirin + PL	Normal mucosa with mild hyperplasia and mild edematous submucosa
